# Altered spontaneous activity in the frontal gyrus in dry eye: a resting-state functional MRI study

**DOI:** 10.1038/s41598-021-92199-8

**Published:** 2021-06-21

**Authors:** Kang Yu, Yu Guo, Qian-Min Ge, Ting Su, Wen-Qing Shi, Li-Juan Zhang, Hui-Ye Shu, Yi-Cong Pan, Rong-Bin Liang, Qiu-Yu Li, Yi Shao

**Affiliations:** 1 Department of Ophthalmology and Radiology, Jiangxi Center of Natural Ocular Disease Clinical Research Center, The First Affiliated Hospital of Nanchang University, No 17, YongWaiZheng Street, DongHu District, Nanchang, 330006 Jiangxi People’s Republic of China; 2 Fujian Provincial Key Laboratory of Ophthalmology and Visual Science, Medical College of Xiamen University, Eye Institute of Xiamen University, Xiamen, 361102 Fujian Province China; 3 Department of Ophthalmology, Massachusetts Eye and Ear, Harvard Medical School, Boston, MA 02114 USA

**Keywords:** Diagnostic markers, Diagnostic markers

## Abstract

This study investigated neurologic changes in patients with dry eye (DE) by functional magnetic resonance imaging (fMRI) and to used regional homogeneity (ReHo) analysis to clarify the relationship between these changes and clinical features of DE. A total of 28 patients with DE and 28 matched healthy control (HC) subjects (10 males and 18 females in each group) were enrolled. fMRI scans were performed in both groups. We carried out ReHo analysis to assess differences in neural activity between the 2 groups, and receiver operating characteristic curve (ROC) analysis was performed to evaluate the performance of ReHo values of specific brain areas in distinguishing DE patients from HCs. The relationship between average ReHo values and clinical characteristics was assessed by correlation analysis. ReHo values of the middle frontal gyrus, inferior frontal gyrus, and superior frontal gyrus were significantly lower in DE patients compared to HCs. The ROC analysis showed that ReHo value had high accuracy in distinguishing between DE patients and HCs (P < 0.0001). The ReHo values of the middle frontal gyrus and dorsolateral superior frontal gyrus were correlated to disease duration (P < 0.05). Symptoms of ocular surface injury in DE patients are associated with dysfunction in specific brain regions, which may underlie the cognitive impairment, psychiatric symptoms, and depressive mood observed in DE patients. The decreased ReHo values of some brain gyri in this study may provide a reference for clinical diagnosis and determination of treatment efficacy.

## Introduction

Dry eye (DE) is characterized by the perturbation of tear film homeostasis and eye discomfort. Common causes include tear film damage, abnormal tear fluid, inflammation, and injury; and the main clinical manifestations are dry eyes, loss of vision, foreign body sensation in the eyes, burning sensation, and photophobia. Epidemiologic studies have revealed a significantly higher incidence of dry eyes in Asian populations than in Europe. The symptoms of dry eyes are often accompanied by neurosensory abnormalities^1–3^, suggesting that the onset of dry eyes can lead to functional abnormalities of the central nervous system. The DE on Daily Life Impact survey demonstrated that DE reduces patients’ social, physical, and spiritual functioning; therefore, the occurrence of DE warrants clinical attention^4^. DE is generally considered to be a corneal disease, but our previous work has shown that some ophthalmologic diseases are accompanied by changes in specific brain areas^5,6^, although the nature and significance of this association are unclear. Pan et al. used Global-brain FC to analyze the images of 20 dry eye patients and found that the difference in the value of left thalamus can distinguish patients from normal people^7^. The study by Levitt et al. also showed that the cause of dry eye pain may be related to abnormal central pain management^8^. At present, early detection and early treatment of DE are very important, and standard treatment strategies can greatly improve the prognosis of patients. Therefore, our research can provide new ideas for the clinical diagnosis of DE. Through the changes of brain regions, DE can be detected as early as possible and appropriate interventions can be carried out. Magnetic resonance imaging (MRI) has enabled the study of brain areas involved in human cognition and the localization and quantification of neuronal activity. Functional (f)MRI is the main modality used to evaluate the functional state of the brain^9^. Compared to traditional MRI technology, fMRI has higher spatial resolution and allows detailed visualization of brain microstructure and changes thereof. fMRI comprises diffusion weighted imaging (DWI) and diffusion tensor imaging (DTI). The former reveals spatial information on tissues and can be used to detect early morphologic and physiologic abnormalities based on changes in tissue water content, while the latter is the only technique that allows 3-dimensional (3D) analysis of white matter fiber bundles based on the direction of diffusion of water molecules. It was reported that gray matter volume in the occipital and parietal eye fields was significantly reduced in patients with strabismus compared to healthy subjects^10^. Additionally, functional abnormalities in multiple brain regions have been reported in patients with eye diseases^11^. However, there have been no studies to date using fMRI to investigate DE.

MRI signals reflect functional networks in the central nervous system^12^; changes in the resting state signal can provide a basis for predicting and diagnosing diseases including primary insomnia^13^, obstructive sleep apnea^14,15^, and sleep deprivation^16^. Analysis of regional homogeneity (ReHo) in resting-state fMRI is a sensitive and reliable method for measuring changes in neural activity^17^. An advantage of ReHo is that it does not require a seed region to be specified, and can therefore be used to detect changes in any brain region^18^. ReHo analysis has been used to investigate the pathogenesis of epilepsy^19^, primary insomnia^13^, Parkinson disease^20^, depression^21^, and other neurologic diseases; and ophthalmologic studies have applied this method to optic neuritis^22^ and comitant strabismus^11^. In the present work, we explored changes in brain activity in DE and examined their relationship to clinical features by fMRI and ReHo analysis.

## Materials and methods

### Subjects

Patients with moderate or severe DE (N = 28; 18 women and 10 men) were recruited at the Ophthalmology Department of the First Affiliated Hospital of Nanchang University Hospital. The following criteria were used to diagnose: dry eyes; foreign body sensation, burning sensation, cracking and itching, photophobia, blurred vision, visual fatigue, and other annoying visual symptoms; variable conjunctival and corneal staining; tear breakup time < 10 s, Schirmer I test < 10 mm/5 min.

**Figure 1:**
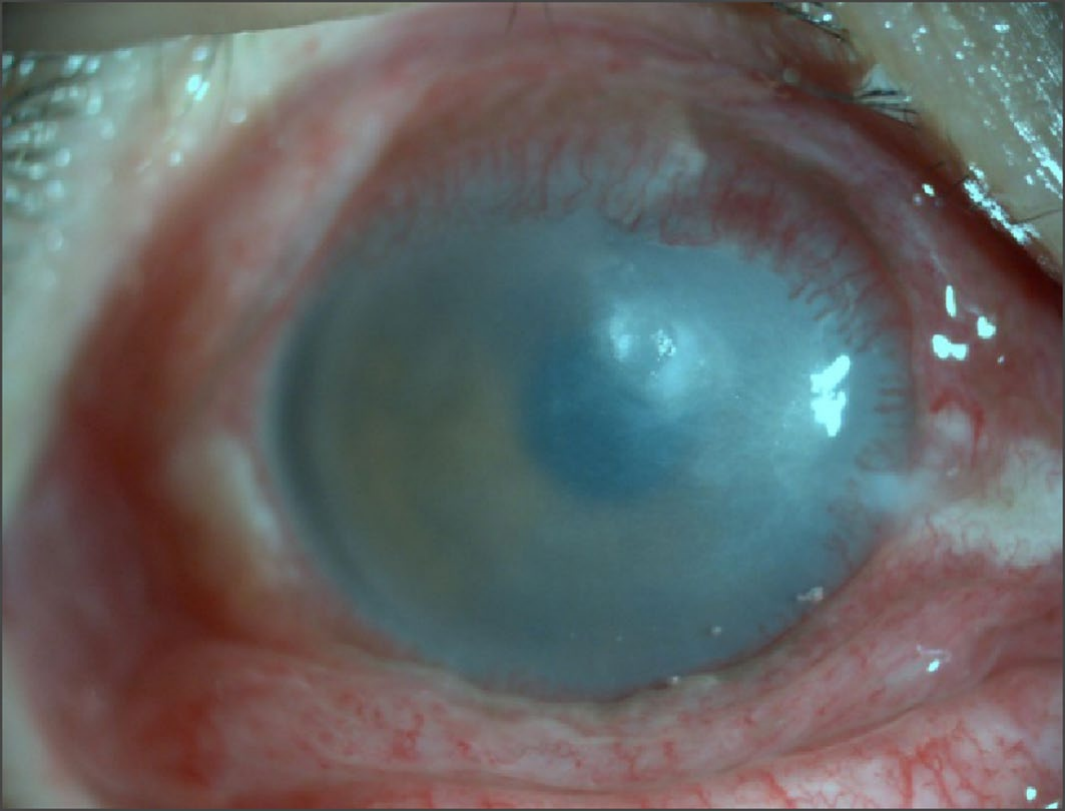
A representative ocular surface photography of severe DE.

Inclusion criteria for DE patients were as follows: (1) met the diagnostic criteria for lacrimal dry eye; (2) age 20–75 years old; (3) not treated with any drugs or had discontinued treatment for > 2 weeks prior to enrollment; and (4) provided informed consent to participate in the study. Exclusion criteria were as follows: (1) conjunctival scarring, atresia of the lacrimal gland opening, or complete atrophy of accessory lacrimal glands; (2) other obvious conjunctiva, cornea, or iris lesions; (3) pregnant or lactating women; (4) suspected or confirmed history of drug abuse (Fig. 1).

We also recruited 28 healthy control (HC) subjects (18 women and 10 men) who were matched to DE patients in terms of age, sex, and other demographic parameters. The HCs met the following criteria: (1) no abnormalities in brain parenchyma observed by MRI; (2) no eye diseases, visual impairment, or corrected vision (visual acuity [VA] > 1.0); (3) normal mental health and no abnormalities upon neurologic examination; and (4) no contraindications for MRI. This study was authorized by the ethics committee of the First Affiliated Hospital of Nanchang University Hospital, and the protocol met the requirements of the Declaration of Helsinki and conformed to the principles of medical ethics. All volunteers participated voluntarily and were informed of the purpose, methods, and potential risks of the study. All participants signed an informed consent form.

### MRI parameters

MRI was performed with a 3-Tesla magnetic resonance scanner (Magnetom Trio; Siemens, Munich, Germany). Subjects were instructed to keep their eyes closed but remain awake and relaxed until the end of the scan. Data were obtained with a 3D spoiled gradient-recalled echo sequence. Imaging parameters of the T1- and T2-weighted image sequences (176 images) were as follows: repetition time (TR) = 1900 ms, echo time (TE) = 2.26 ms, thickness = 1.0 mm, gap = 0.5 mm, acquisition matrix = 256 × 256, field of view = 250 × 250 mm, and flip angle = 9°^5^. Imaging parameters for the 240 functional images were as follows: TR = 2000 ms, TE = 30 ms, thickness = 4.0 mm, gap = 1.2 mm, acquisition matrix = 64 × 64, flip angle = 90°, field of view = 220 × 220 mm, and 29 axials. Scanning times were 5 and 10 min, respectively.

### fMRI data processing

MRI data of eligible patients were collected during their hospitalization, from September 2019 to March 2020. MRIcro software (https://people.cas.sc.edu/rorden/mricro/mricro.html) was used to filter and classify the acquired data. To stabilize the scan signal, the first 15 scanned images were discarded. Data Processing Assistant for Resting-State fMRI v4.0 software (http://rfmri.org/DPARSF) and Statistical Parametric Mapping software (http://www.fil.ion.ucl.ac.uk/spm) were used for data preprocessing; the main steps were slice timing; head motion correction using Friston-6 head motion parameters to regress out head motion effects; spatial normalization with standard echo planar image templates to achieve Neurology Montreal Institute standards; and smoothening with a Gaussian kernel of 6 × 6 × 6 mm^3^ full-width at half-maximum. REST software (State Key Laboratory of Cognitive Neuroscience and Learning, Beijing Normal University, Beijing, China) was used to calculate ReHo by analyzing Kendall consistency coefficients of a given voxel and the adjacent voxel time series.

### Statistical analysis

Differences in demographic and clinical data between DE patients and HCs were evaluated with the independent samples t-test using SPSS v20.0 software (SPSS, Chicago, IL, USA) differences in ReHo values between SA and HC subjects were evaluated using two-sample t-tests in REST software (State Key laboratory of cognitive neuroscience and learning, Beijing Normal University, Beijing, China). At the voxel level, the statistical threshold was set to P < 0.05, and for multiple comparisons using Gaussian random field theory voxels, thresholds of P < 0.001 and cluster size of > 40 voxels (AlphaSim-corrected) were employed. We speculated that differences in ReHo values can serve as a biomarker for diagnosing DE and identifying any associated neurologic abnormalities, and tested this hypothesis by receiver operating characteristic (ROC) curve analysis. Accuracy was considered low or high if the area under the ROC curve (AUC) was 0.5–0.7 and 0.7–0.9, respectively. For all tests, statistical significance was set at P < 0.05.

### Brain-behavior correlation analysis

REST software divides the brain into different regions of interest (ROIs) based on different ReHo values. The average ReHo value for each ROI is the average of all ReHo voxels for that region. Correlations between ReHo values in ROIs and clinical features of DE patients were evaluated by Pearson’s correlation analysis; a difference with a P value < 0.05 was considered statistically significant.

## Results

### Demographic information and visual measurements

There were no significant differences in age (P = 0.839), height (P = 0.668), body weight (P = 0.724), body mass index (P = 0.912), or best-corrected same-eye VA between DE patients and HCs (Table 1).

**Table 1:** Clinical characteristics of patients between DE and HC groups.

Characteristics	DE	HCs	*t*-value	*p*-values
Male/female	10\18	10\18	NA	NA
Age (years)	56.13 ± 9.72	55.23 ± 9.18	− 0.238	0.839
Weight(kg)	57.24 ± 7.36	58.24 ± 6.97	− 0.412	0.724
Height (cm)	165.53 ± 9.28	164.32 ± 6.16	− 0.418	0.668
BMI (kg/m^2^)	22.61 ± 1.54	21.93 ± 1.46	− 0.049	0.912
Duration of DED (mons)	9.67 ± 3.14	NA	NA	NA
Duration from onset of DED to rs-fMRI scan (mons)	9.42 ± 2.98	NA	NA	NA
Best-correted VA, right	0.95 ± 0.35*	1.15 ± 0.15	− 0.512	0.538
Best-correted VA, left	0.85 ± 0.40*	1.05 ± 0.20	− 0.648	0.659

Independent t-tests comparing the two groups (*P < 0.05) represented statistically significant differences).

*DE* dry eye, *HCs* healthy controls, *NA* not applicable, *BMI* body mass index, *rs-fMRI* resting-state functional magnetic resonance, *VA* visual acuity.

### ReHo differences

**Figure 2:**
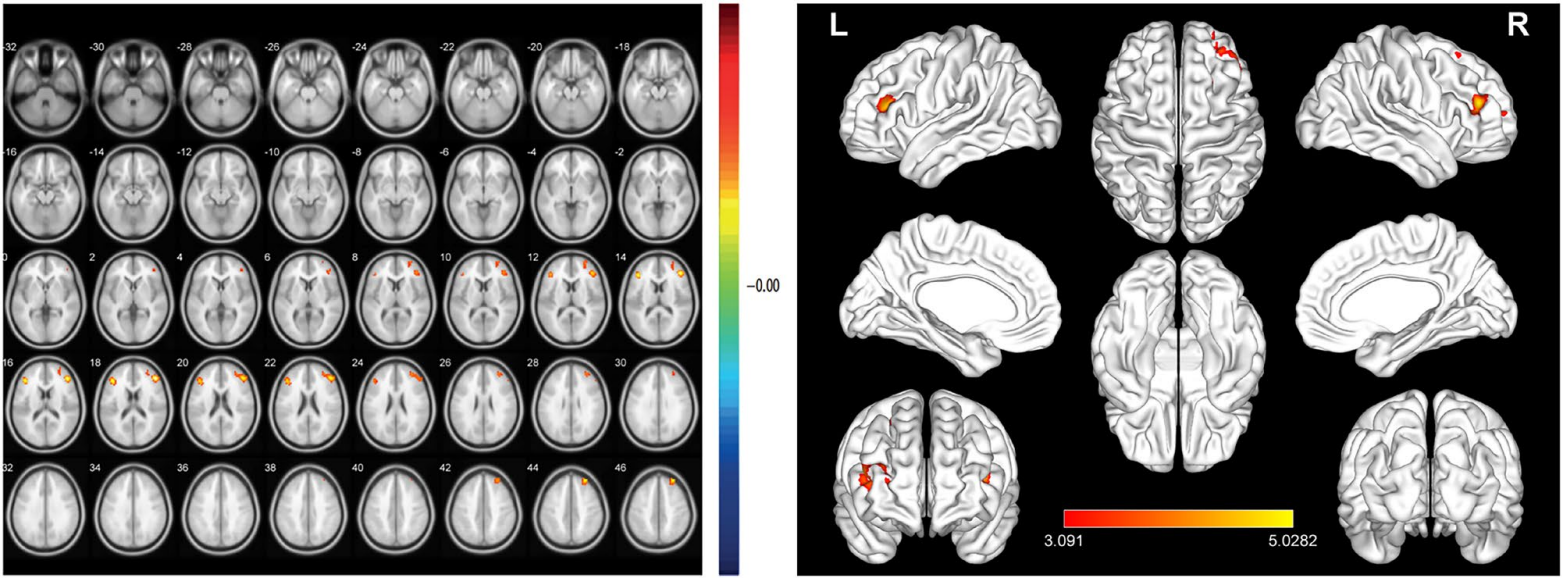
Mean ReHo values of altered brain regions. Compared to HC subjects, the ReHo values of the following regions were decreased in DE patients: triangular part of the IFG (IFGtriang.L; t = 4.7284), MFG (MFG.R; t = 5.0282), and dorsolateral SFG (SFGdor.R; t = 4.4802). *BA* Brodmann’s area.

ReHo values of the triangular part of the inferior frontal gyrus (IFG), middle frontal gyrus (MFG), and dorsolateral superior frontal gyrus (SFG) were significantly lower in DE patients than in HC subjects (Fig. 2 and Table 2).

**Table 2:** Brain areas with significantly different ReHo values between groups.

Condition	Left/right	Brain areas	MNI coordinates			number of voxels	t-value
			X	Y	Z		
		**HC > DE**					
1	Right	Middle frontal gyrus	45	39	18	191	5.0282
2	Left	Inferior frontal gyrus, triangularpart	− 42	33	15	73	4.7284
3	Right	Superior frontal gyrus dorsolateral	30	45	45	72	4.4802

The statistical threshold was set at voxel level with P < 0.05 for multiple comparisons using Gaussian random field theory voxels with P < 0.001 and cluster size > 40 voxels, alphaSim corrected.

*ReHo* regional homogeneity, *DE* dry eye, *HC* healthy control, *MNI* Montreal neurological institute.

**Figure 3:**
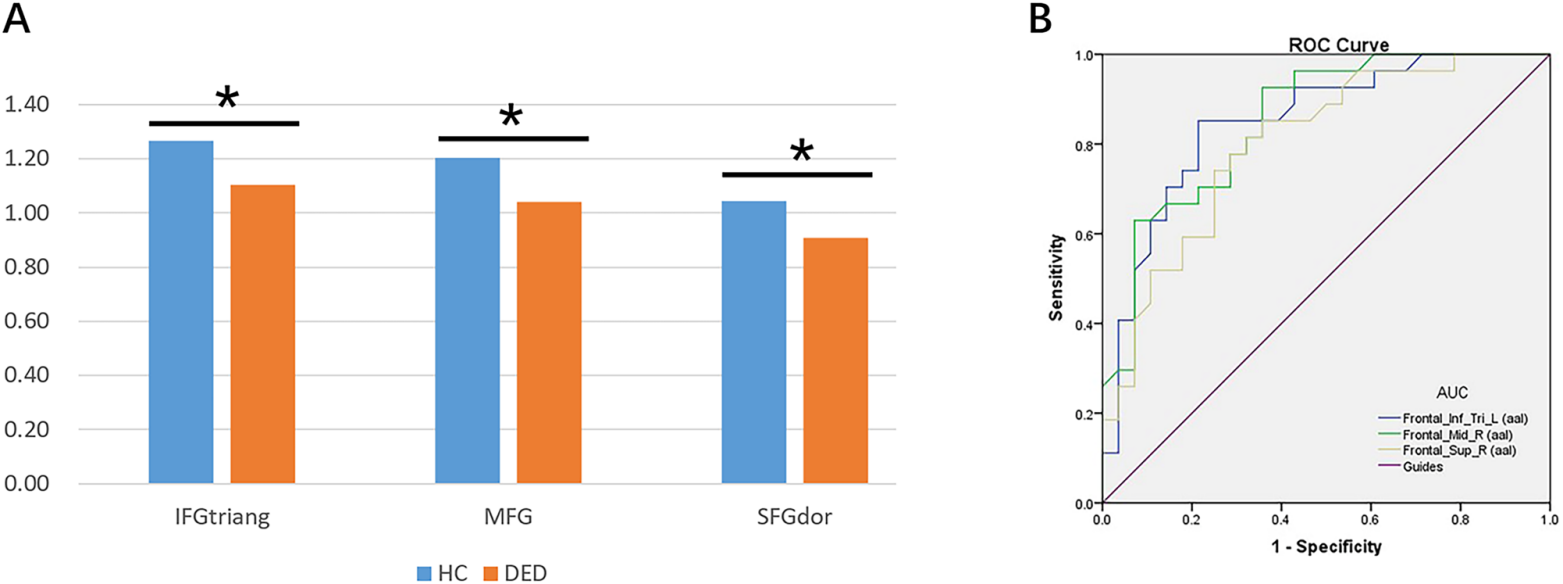
(**A**) The average ReHo value of DE and HCs. IFGtriang: Inferior frontal gyrus triangularpart; MFG: Middle frontal gyrus; SFGdor: Superior frontal gyrus dorsolateral. (**B**) ROC Analysis of ReHo Value of DE. *Inf_Tri_L* Inferior frontal gyrus triangularpart, *Mid_R* Middle frontal gyrus, *Sup_R* Superior frontal gyrus dorsolateral, *ROC* Receiver operating characteristic curve, *AUC* Area Under Curve.

**Figure 4:**
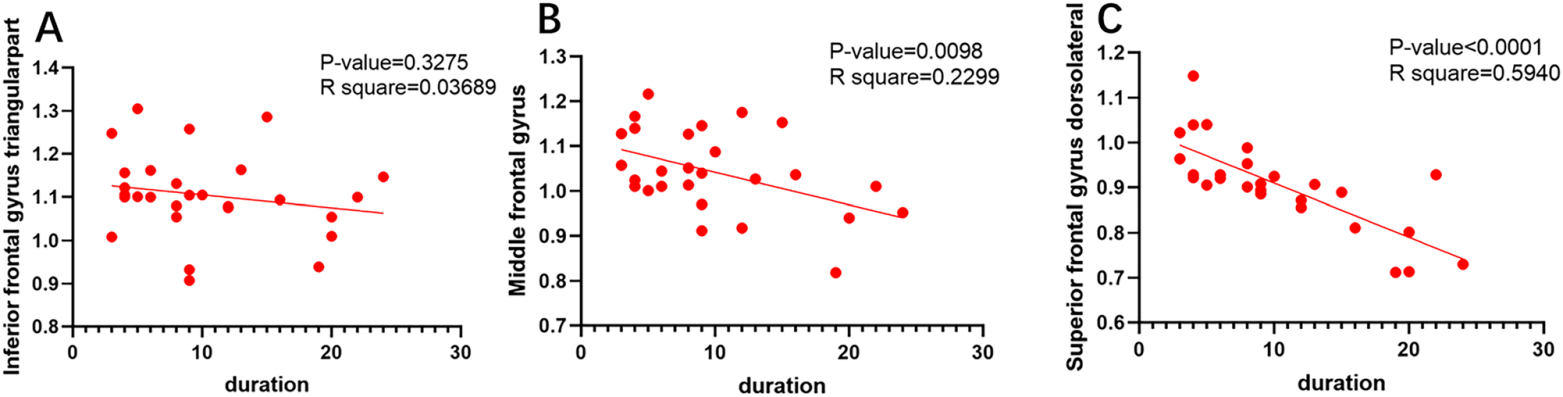
The correlation analysis between the course of the disease and the average ReHo of the brain area. (**A**) is the correlation between the ReHo value of awkward gyrus triangularpart and duration of disease (R square = 0.03689, P = 0.3275). (**B**) is the correlation between the ReHo value and duration of disease (R square = 0.2299). The P value is 0.0098. (**C**) shows the correlation between the ReHo value and duration of disease, R square = 0.5940, and P < 0.0001.

The average ReHo values of the 2 groups are shown in Fig. 3A. The values of the MFG and SFG showed an inverse correlation with disease duration. We found that there was a correlation between patient ReHo values and progression duration, and middle and Superior beta gyrus Dorsolateral ReHo values were correlated with progression duration, which was statistically significant. (P < 0.05; Table 3, Fig. 4).

**Table 3:** Pearson correlations analysis.

Brain regions	ReHo value (mean ± SD)	Duration (years) (mean ± SD)	r-value	P-value
Inferior frontal gyrus triangular part	1.1045 ± 0.0964	9.67 ± 3.14	0.03689	0.3275
Middle frontal gyrus	1.0399 ± 0.0933	9.67 ± 3.14	0.2299	0.0098
Superior frontal gyrus dorsolateral	0.9071 ± 0.0968	9.67 ± 3.14	0.5940	< 0.0001

*ReHo* regional homogeneity, *SD* standard deviation.

Compared with HC, the ReHo values of the inferior frontal gyrus triangularpart, middle frontal gyrus, and superior frontal gyrus dorsolateral areas of DE patients were significantly reduced.

### ROC curve analysis

Given the abnormalities in the activity of certain brain regions in DE patients detected by fMRI, we investigated whether ReHo value has diagnostic utility for DE by ROC curve analysis. The AUCs were 0.85 (95% confidence interval [CI] 0.747–0.953; P < 0.0001) for the triangular part of the IFG, 0.853 (95% CI 0.754–0.951; P < 0.0001) for the MFG, and 0.802 (95% CI 0.687–0.918; P < 0.0001) for the dorsolateral SFG (Fig. 4), indicating that the ReHo values of these brain areas have high good accuracy in distinguishing DE patients from HCs. See Fig. 3B.

## Discussion

DE is a disease in which tear film instability or ocular surface damage caused by changes in the quantity and quality of tear fluid or abnormal tear fluid dynamics results in ocular discomfort and visual dysfunction. As an early complication after corneal refractive surgery, DE can usually be cured 6–9 months after surgery. DE has a multifactorial etiology and is often accompanied by mental or neurologic disorders such as depression, anxiety, stress, posttraumatic stress disorder, and sleep disorders. Drugs used to treat mental illnesses can also affect DE along with neuropathic pain, chronic pain syndrome, peripheral neuropathy, and central nervous system diseases^23^. Thus, DE is likely related to nervous system and brain dysfunction.

Resting-state functional magnetic resonance imaging can provide insight into brain abnormalities in disease states^24^. The ReHo method can reveal abnormal activities in specific brain regions^25^. At present, the ReHo method has been successfully applied to a variety of craniocerebral injuries, neurogenic diseases and ophthalmological diseases, with great potential for development (Table 4). As far as we know, the current study is the first to use ReHo technology to evaluate resting brain activity in DE patients.

**Table 4:** Application of ReHo in ophthalmology and other diseases.

Author	Year	Disease	Refs.
**Ophthalmological diseases**			
Song et al.	2014	Glaucoma	^26^
Cui et al.	2014	Diabetic retinopathy	^27^
Shao et al.	2015	Optic neuritis	^28^
Xu et al.	2019	Corneal ulcer	^29^
Xiang et al.	2019	Trigeminal neuralgia	^30^
Liao et al.	2019	Diabetic retinopathy	^31^
**Neurogenic diseases**			
Dai et al.	2012	Sleep disorders	^32^
Li et al.	2016	Parkinson's disease	^33^

**Figure 5:**
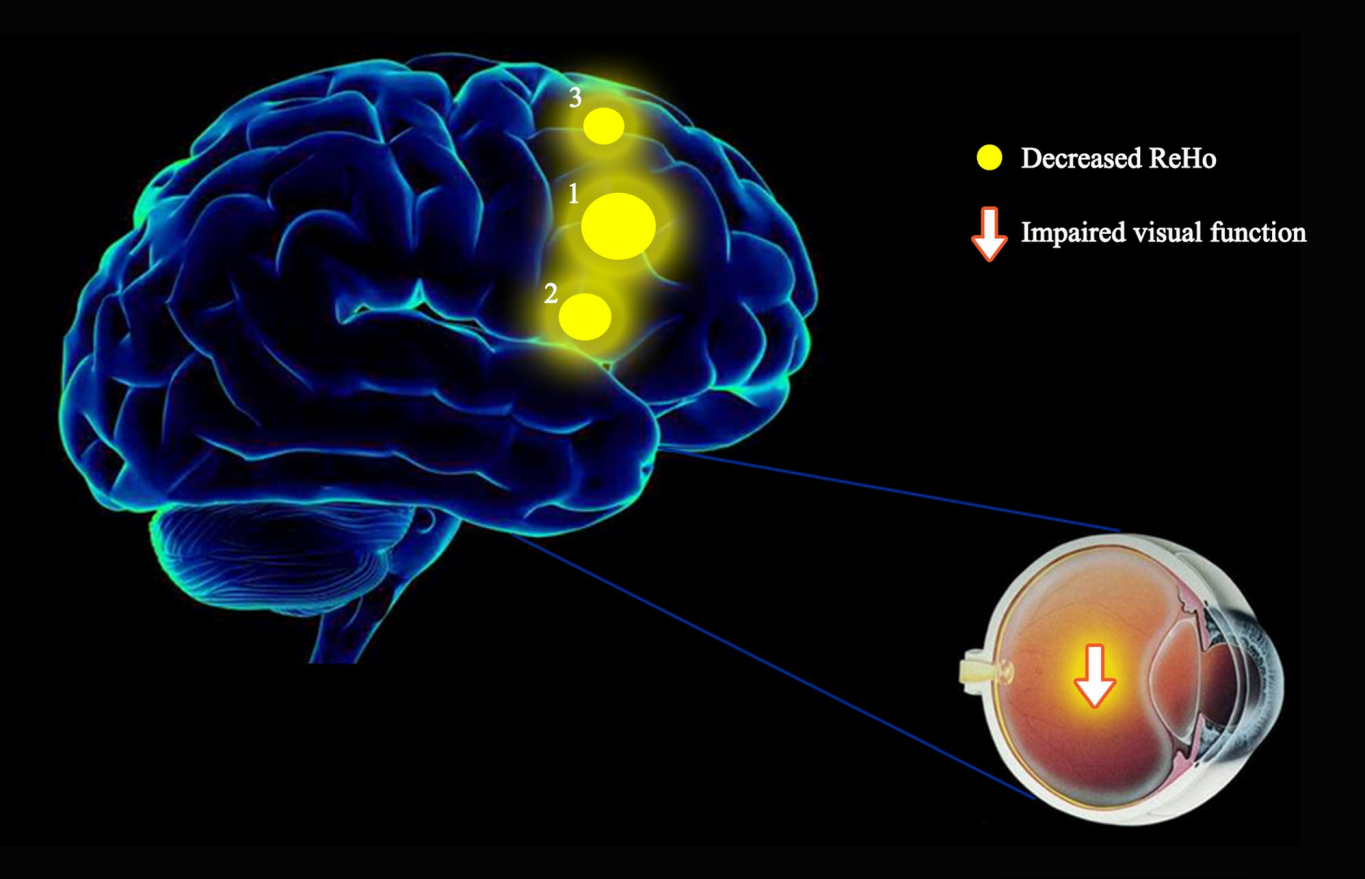
The mean ReHo values of altered brain regions. Compared with the HCs, the ReHo values of the following regions were decreased to various extents: (1) Middle frontal gyrus (t = 5.0282), (2) Inferior frontal gyrus triangular part (t = 4.7284), (3) Superior frontal gyrus dorsolateral (t = 4.4802). *HCs* healthy controls, *BA* Brodmann's area.

Compared with HC, the ReHo values of the inferior frontal gyrus triangularpart, middle frontal gyrus, and superior frontal gyrus dorsolateral areas of DE patients were significantly reduced. Figure 5 shows the abnormal brain areas.

The MFG is located in the frontal lobe of the cerebral cortex between the suprafrontal and subfrontal sulci; the posterior part of the MFG processes information related to the movement of the head and eyes. The MFG has been implicated in response to stress^33^ and cognitive function^34^, and we previously showed that it is more active during task performance^35^. A study using DTI to analyze structural changes in the brain of patients with ophthalmologic diseases showed that the diffusion coefficient of the MFG was significantly lower in these individuals than in control subjects^36^.

The IFG is mainly involved in language and voluntary movement and is implicated in the neurologic effects associated with ocular surface damage in DE patients. We found that the ReHo value of the IFG in DE patients was significantly lower than that of HCs, which is consistent with our previous research^5^.

The SFG is linked to depression and plays an important role in cognition and attention^37^. Abnormalities in the SFG have been observed in patients with depression ^38–40^. Interestingly, depression has been reported in patients with DE and other corneal diseases^41,42^. We speculate that this is related to a decrease in the ReHo value of the SFG, which was observed in our study. Our research results show that the clinical symptoms of DED patients are indeed related to brain dysfunction, which is consistent with the study of Alexandra et al.

**Figure 6:**
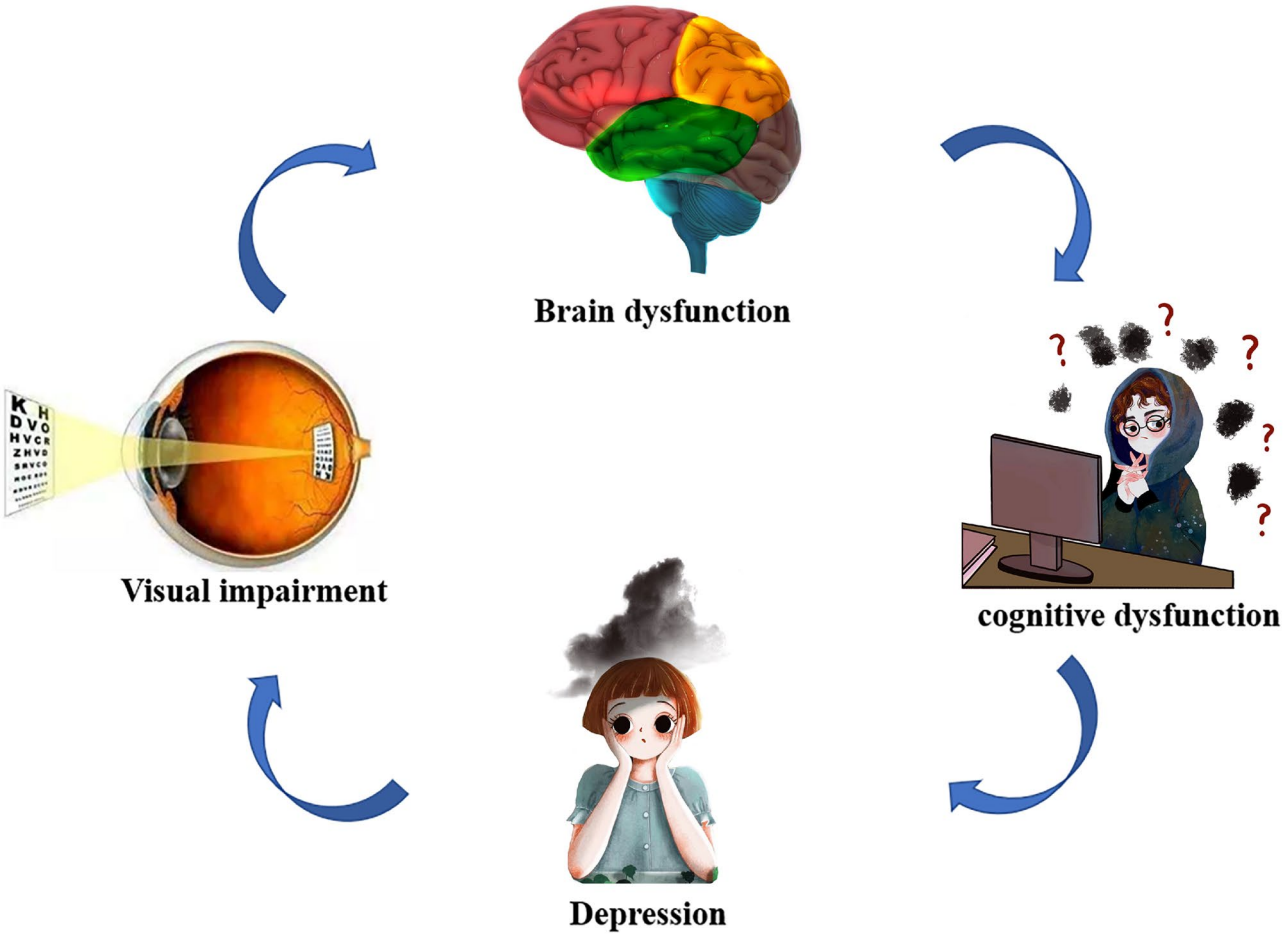
Relationship between MRI images and clinical manifestations in DE. *MRI* magnetic resonance imaging, *DE* dry eye.

Therefore, we speculate that DE may cause brain dysfunction. (Fig. 6, Table 5). This study also used the ROC curve, and the results showed that the AUC of each brain area was greater than 0.7. These REHO values can be used to supplement the results of statistical analysis, but the ReHo values cannot provide diagnostic capabilities.

**Table 5:** Brain regions alternation and its potential impact.

Brain regions	Experimental	Brain function	Anticipated results
Middle frontal gyrus	DEs < HCs	Key parts of word processing and processing	Cognitive activity disorder
Inferior frontal gyrus	DEs < HCs	Visual processing, emotional processing	Visual impairment, dementia, etc.
Superior frontal gyrus	DEs < HCs	Cognitive, emotional, pain, and behavioral management	Irritability, mood swings, depression, etc.

*DE* dry eye, *HC* healthy control.

This study had some limitations. Firstly, the sample size was small; additional studies are needed in a larger population in order to validate our findings. Second, the length of the scan time and small body movements may affect the scan results; these individual differences may undermine the accuracy of our analysis.

In addition, the literature shows that there is no spatial smoothing in data preprocessing to improve the reliability of ReHo^43^, and the 6 × 6 × 6 mm smoothing method we use may have certain errors.

Nonetheless, the results of our study demonstrate that DE patients exhibit abnormal neural activity in the frontal gyrus, which may be related to DE pathogenesis. The ReHo values of the IFG, MFG, and SFG can be used to assist clinicians in identifying DE and help judge the prognosis.
