# Author Correction: Altered spontaneous activity in the frontal gyrus in dry eye: a resting-state functional MRI study

**DOI:** 10.1038/s41598-021-97182-x

**Published:** 2021-08-30

**Authors:** Kang Yu, Yu Guo, Qian-Min Ge, Ting Su, Wen‑Qing Shi, Li‑Juan Zhang, Hui‑Ye Shu, Yi‑Cong Pan, Rong‑Bin Liang, Qiu‑Yu Li, Yi Shao

**Affiliations:** 1 Department of Ophthalmology and Radiology, Jiangxi Center of Natural Ocular Disease Clinical Research Center, The First Affiliated Hospital of Nanchang University, No 17, YongWaiZheng Street, DongHu District, Nanchang, 330006 Jiangxi People’s Republic of China; 2 Fujian Provincial Key Laboratory of Ophthalmology and Visual Science, Medical College of Xiamen University, Eye Institute of Xiamen University, Xiamen, 361102 Fujian Province China; 3 Department of Ophthalmology, Harvard Medical School, Massachusetts Eye and Ear, Boston, MA 02114 USA

Correction to: *Scientific Reports*, 10.1038/s41598-021-92199-8, published online 21 June 2021

The original version of this Article contained an error in the spelling of the author Qian-Min Ge which was incorrectly given as Qian-Ming Ge.

The original Article has been corrected.

